# Novel indolyl-chalcone derivatives inhibit A549 lung cancer cell growth through activating Nrf-2/HO-1 and inducing apoptosis *in vitro* and *in vivo*

**DOI:** 10.1038/s41598-017-04411-3

**Published:** 2017-06-20

**Authors:** Xuan Zhao, WenLiang Dong, YuanDi Gao, Dong-Shoo Shin, Qing Ye, Le Su, Fan Jiang, BaoXiang Zhao, JunYing Miao

**Affiliations:** 1 Shandong Provincial Key Laboratory of Animal Cells and Developmental Biology, School of Life Science, Shandong University, Jinan, 250100 China; 2 Department of Chemistry, Changwon National University, Changwon, 51140 South Korea; 3 The Key Laboratory of Cardiovascular Remodeling and Function Research, Chinese Ministry of Education and Chinese Ministry of Health, Qilu Hospital, Shandong University, Jinan, 250012 China; 4 Institute of Organic Chemistry, School of Chemistry and Chemical Engineering, Shandong University, Jinan, 250100 China

## Abstract

Increasing evidence indicates that Nrf-2, named the nuclear factor-erythroid 2-related factor, may perform anticancer function. In this study, a series of novel substituted phenyl- (3-methyl-1H-indol-2-yl)-prop-2-en-1-one (indolyl-chalcone) derivatives were synthesized and their effects on Nrf-2 activity were observed. We found that compounds 3a**-3d** and 6c elevated Nrf-2 activity. Then we evaluated their anticancer activities *in vitro* and *in vivo* by utilizing human lung cancer cell line A549. The *in vitro* results showed that among the compounds, **3d** performed effectively anti-growth activity by inducing A549 lung cancer cell apoptosis and activating Nrf-2/HO-1 (heme oxygenase-1) pathway. *In vivo*, we proved that compound **3d** inhibited the tumor growth effectively through inducing cell apoptosis without affecting CAM normal angiogenesis. These data suggest that our discovery of a novel Nrf-2 activator compound **3d** would provide a new point of human lung cancer treatment.

## Introduction

Lung cancer is a considerable worldwide public health concern. Comparing with the survival rates of other cancers, 5-years survival rate of lung cancer is lower^1^. Therefore, much more attention has been paid to the discovery of new anticancer drugs^2^.

Cytotoxicity induced by xenobiotic can cause cell death^3^. It has been reported that cell death plays a crucial role in the progress of cancer. As we know, apoptosis and programmed necrosis are two major types of cell death which show different cell morphologies and pathways. Furthermore, as a new alternative target in tumor treatment, autophagy needs us to pay more attention^4^. Discovering new agents in anticancer therapy by inducing different cell death types is meaningful.

Chalcones, are diffusely existing in natural plant products. It has been reported that chalcones have many pharmacological and biological activities, such as anti-oxidative, anti-cancer, anti-mutagenic, anti-inflammatory, etc. The biological activities of chalcones maybe changed through the interaction with different compounds^5^. There are a lot of chalcone derivatives that have been synthesized and identified by researchers in the laboratory through different chemical methods^6^. Many reports have displayed that minor structural transformation of chalcones could induce considerable difference in the effect of anticancer, anti-inflammatory or autoimmune diseases^6, 7^. For example, compound II2, a novel dithiocarbamate–chalcone derivative could apparently inhibit the growth of SK-N-SH cells by triggering apoptosis and blocking the cell cycle^8^. Chalcones could prevent cancer by inhibiting p53 degradation^9^. In human breast cancer, Cathepsin-K contributes to tumor spread. Chalcones agents can suppress Cathepsin-K enzyme activity and effectively inhibit tumor invasiveness in body^10^. As we know that PI3K/Akt/mTOR pathway which modulates cell proliferation, metabolism, apoptosis, autophagy and other cellcular biological activities is important in tumorigenesis^11, 12^. A novel quinazolinone chalcone derivative (QC) has been reported to inhibit PI3K/Akt/mTOR signaling pathway and trigger human HCT-116 cells apoptosis^13^. A pyrrole derivative of chalcone, (*E*)- 3-phenyl-1-(2-pyrrolyl)-2-propenone (PPP) performs anti-inflammatory effect through inhibiting the activity of Syk, Src, and TAK1^14^. It has been also reported that the dysfunction or abnormal proliferation of immune cells may cause autoimmune diseases, atherosclerosis, and tuberculosis^15, 16^. Chalcones as immunomodulatory drugs show different effects on various immune cells, including triggering apoptosis in dendritic cells^17^, inhibiting superoxide anion production by weakening the activity of PKC in PMA-induced rat neutrophils^18^, performing anti-inflammatory potential in monocytes and macrophages^19^, suppressing rabbit platelets aggregation caused by arachidonic acid or collagen^20, 21^, inhibiting the generation of functional cytotoxic T cells from mouse spleen and so on^22^.

Indole is anther important chemical group in this series of compounds which we have synthesized. It has been reported that indole is known as its heterocyclic system, it involves in the protein synthesis in the form of tryptophan^23^. Indole alkaloids with biological activity are plentiful in the nature, such as strychnine and lysergic acid diethylamide. Various indole alkaloids isloated from plants have been reported for some therapeutic value, including anticancer, the treatment of Hodgkin’s diseases^24^ and psychiatric disorders^25^, anti-inflammatory, cytotoxicity, antiviral^26^, being as antimicrobial agents^27^ and so on. Therefore, indole derivatives have attracted many researchers’ attention and a lot of indole derivatives have been synthesized or extracted from natural resources^28^.

It has been reported that chalcones or indoles have a lot of medicinal value in the treatment of diseases, and various chalcones or indole derivatives have been synthesized. However, to date, few indolyl-chalcone compounds have been reported. Novel indoly-chalcone derivatives (CITs) have been synthesized and identified to play a role in anti-cancer treatment through inducing cell death and inhibiting proliferation in PC3, A549, CLR2119 and PAN02 cells^29^. α-Cyano bis (indolyl) chalcone inhibits the growth of A549 lung cancer cells effectively by promoting tubulin polymerization^30^.

As we know, Nrf-2 (nuclear factor-erythroid 2-related factor) encoded by NFE2L2 (nuclear factor, erythroid 2 like 2) gene belongs to the basic-leucine zipper (bZIP) family of transcription factors and is expressed in various tissues^31^. Nrf-2 maintains cellular redox balance by Kelch-like ECH-associated protein 1 (Keap1)-Nrf-2-antioxidant response element (ARE) pathway to make response to endogenous and exogenous stresses^32^. It has been reported that oxidative stress involves in the initiation of cancer, Nrf-2 might exert anticancer function and is implicated in chemoprevention. For example, as an Nrf-2 activator, the natural product sulforaphane (SFN) that presents in cruciferous vegetables has been researched in clinical trials of different cancers^33^. Moreover, a recent study shows that cisplatin as a well-known anticancer drug has been proved to induce oxidative damages and cell death in Hep-2 cells through improving the expression of Nrf-2 and HO-1. According to the current studies, intensifying Nrf-2 activity seems to be an attractive strategy in the process of cancer treatment.

Here, we synthesized a novel series of indolyl-chalcone derivatives and identified a new Nrf-2 activator named indolyl-chalcone derivative **3d**, which dramatically inhibited tumor growth *in vitro* and *in vivo* by inducing A549 lung cancer cell apoptosis and activating Nrf-2/HO-1 pathway. Additionally, compound **3d** did not show any significantly autophagy or necrosis in A549 lung cancer cells. Consequently, the discovery of the novel Nrf-2 activator would provide a new point of human lung cancer therapy.

## Chemistry: synthesis of compounds

**Figure 1:**
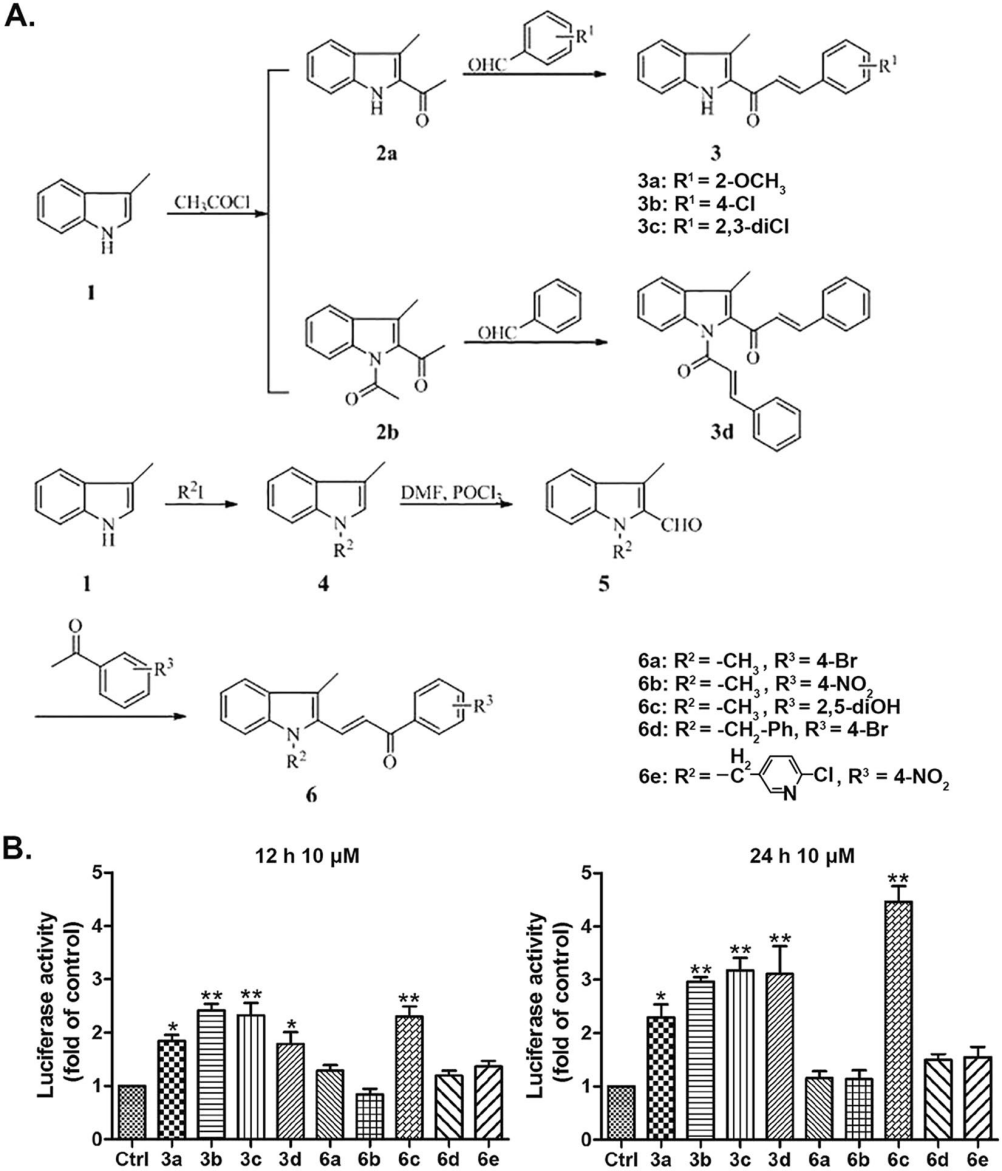
Effects of compounds 3a-3d, 6a-6e on Nrf-2 activity. Chemical structures of compounds 3a-**3d**, 6a-6e (**A**). HeLa cells which contain Nrf-2-responsive/pGL4-3 × ARE-basic luciferase reporter vector were treated with compounds 3a-**3d**, 6a-6e at 10 μM for 12 h or 24 h. The control group (Ctrl) was treated with 0.1% DMSO (V/V). Luciferase activity was determined by luciferase assay, and normalized to cell viability measured by SRB assay. Results are mean ± SEM (*p < 0.05, **p < 0.01 vs control. N = 3).

A series of indolyl-chalcone derivatives were designed and synthesized (Fig. 1A). Compound 2 was synthesized according to the reported method^34^. Compound 3 was synthesized from compound 2. Firstly, the mixture of compound 2 (1 mmol) and NaOH (2 mmol) in ethanol (10 ml) were stirred at room temperature, and then substituted aldehyde (1.2 mmol) in ethanol (5 ml) was added dropwise. The mixture was stirred for 2–4 h. The end of reaction was detected by TLC. Then the mixture was poured into cold water and filtered. The crude product was recrystallized from ethanol to obtain compound 3 in 60-90% yield. Compounds 4 and 5 were synthesized according to previous reported method^35, 36^. Compound 6 was procured by the following reactions. The mixture of substituted acetophenone (1 mmol) and NaOH (2 mmol) in ethanol (10 ml) were stirred at room temperature, and then compound 5 (1.1 mmol) in ethanol (5 ml) was added dropwise. The mixture was stirred for 2–4 h. The end of reaction was detected by TLC. Then the mixture was poured into cold water and filtered. The crude product was recrystallized from ethanol to obtain compound 6 in 60–90% yield.

## Results

### Indolyl-chalcone derivatives (3a-3d, 6c) activate Nrf-2 significantly

As Nrf-2 activators have shown much incredible potential in disease prevention^33^, especially in cancer treatment, we firstly analyzed endogenous Nrf-2 activity in HeLa cells which were transfected with luciferase-based Nrf-2 reporter plasmid after treatment with a series of novel substituted phenyl-(3-methyl-1H-indol-2-yl)-prop-2-en-1-one, indolyl-chalcone derivatives (3a -**3d**, 6a-6e). The luciferase assay suggested that compounds 3a, 3b, 3c, **3d** and 6c (10 μM) elevated Nrf-2 activity significantly compared with the control after treatment for 12 h or 24 h (Fig. 1B).

### Compounds 3c, 3d, 6a-6c inhibit the growth of A549 lung cancer cells at low IC50 values

**Figure 2:**
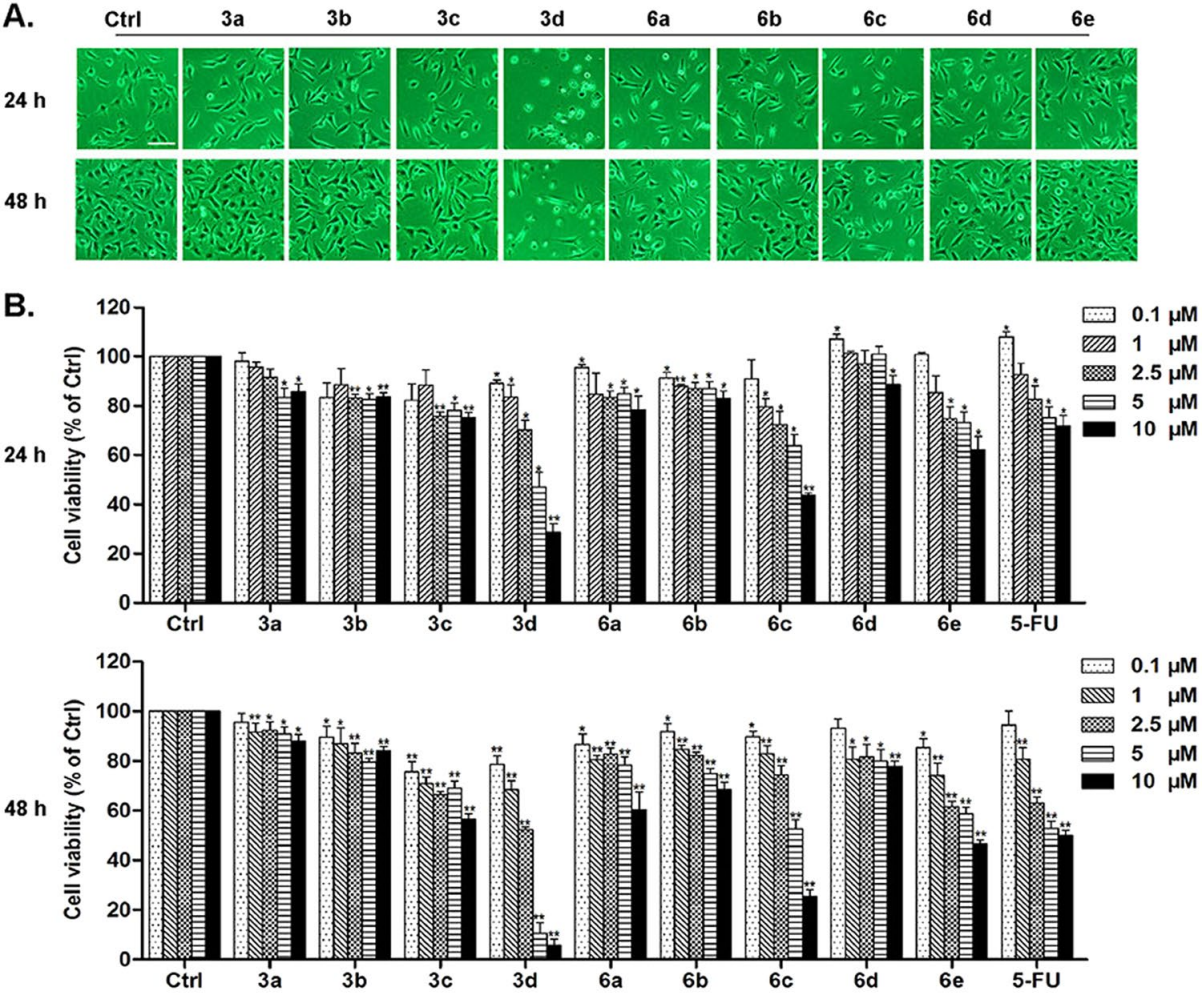
Effects of compounds 3a-3d, 6a-6e on morphology and viability of A549 lung cancer cells. A549 lung cancer cells were treated with compounds 3a-**3d**, 6a-6e (2.5 μM) or 0.1% DMSO (control) for 24 h or 48 h (**A**). Microscopic photographs (200×) were taken by using the inverted phase contrast microscope (Nikon). Scale bar: 20 μm. A549 cells were treated with compounds 3a-**3d**, 6a-6e at 0.1, 1, 2.5, 5, 10 (μM) for 24 h or 48 h (**B**). The control group (Ctrl) was treated with 0.1% DMSO (V/V). Cell viability was analyzed by SRB assay. 5-FU was utilized as a positive drug control. Results are mean ± SEM (*p < 0.05, **p < 0.01 vs control. N = 3).

In order to find out how these compounds influenced tumor cells growth as Nrf-2 activators, we selected A549 lung cancer cells for the following research. We firstly observed the morphological changes of A549 lung cancer cells after treatment with the compounds 3a-**3d**, 6a-6e for 24 h or 48 h by using a phase contrast microscope to investegate the anti-cancer activity of the compounds (Fig. 2A). There was no remarkable morphological change of A549 lung cancer cells treated with the compounds at the dose of 2.5 μM except for compounds **3d** and 6c. Comparing with control group, the cell density reduced in response to the treatment of these compounds. Additionally, we observed that morphology of A549 lung cancer cells significantly shrinked, bleb protrusions formed in the cell membrane and apoptosis body released after treatment with compound **3d** and 6c. Sulforhodamine B (SRB) assay suggested that compound **3d** inhibited the growth of A549 lung cancer cells most efficiently (Fig. 2B, Table 1).

**Table 1:** The IC_50_ values (48 h) of compounds 3a-**3d**, 6a-6e and 5-FU in A549 lung cancer cells.

Compounds	3a	3b	3c	3d	6a
**IC**_**50**_ **(μM)**	**35.78**	**49.35**	**14.03**	**2.46**	**13.56**
**Compounds**	**6b**	**6c**	**6d**	**6e**	**5-FU**
**IC**_**50**_ (**μM)**	**15.12**	**6.02**	**26.44**	**8.09**	**8.21**

### Apoptosis assay of compounds 3c, 3d, 6c in A549 lung cancer cells

**Figure 3:**
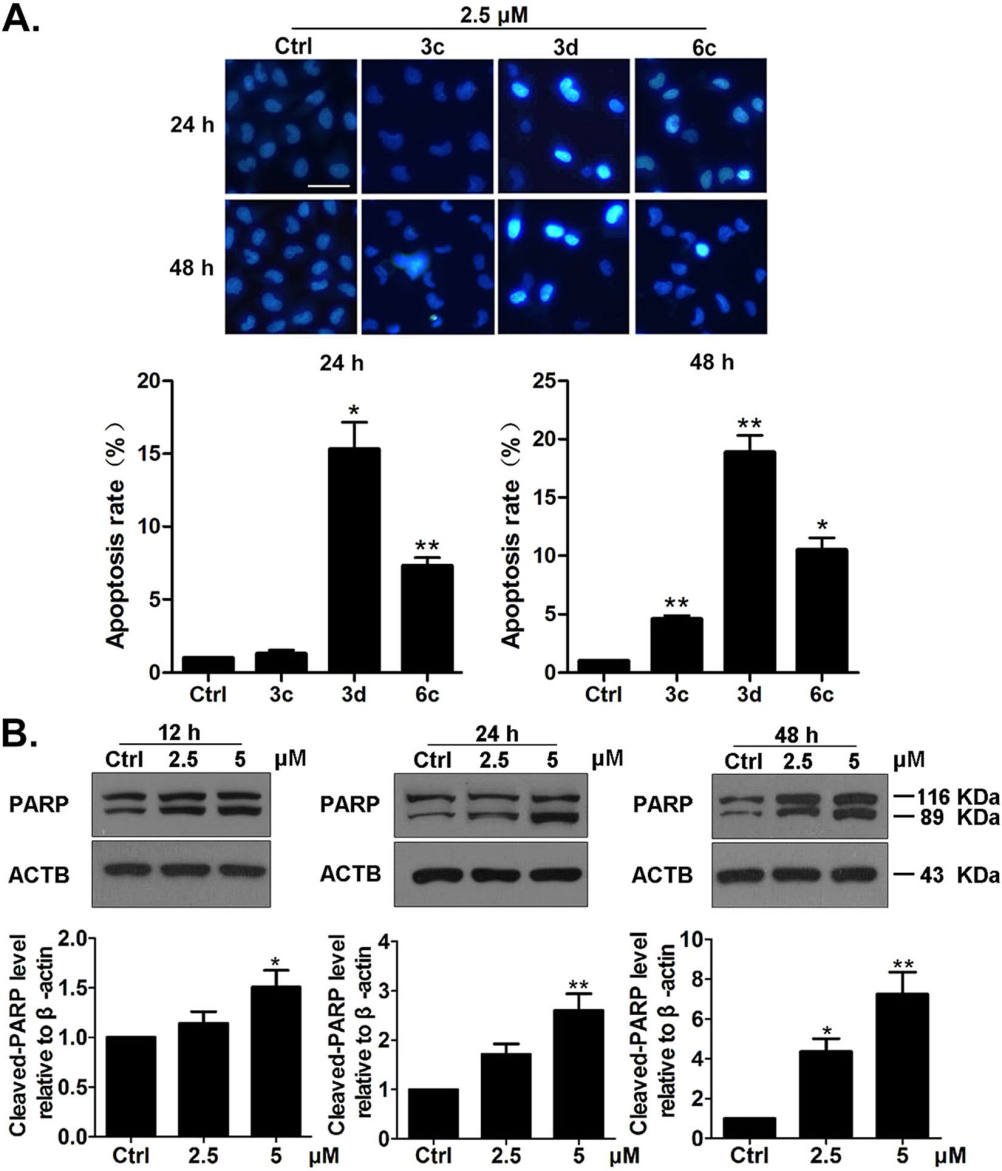
Compounds 3c, **3d** and 6c induce A549 lung cancer cell apoptosis. Hoechst 33258 staining was conducted after treating the cells with the compounds (3c, **3d** and 6c) at 2.5 μM for 24 h and 48 h (magnification 200×). Fluorescent image of A549 lung cancer cells stained by Hoechst 33258 (**A**). Scale bar: 20 μm. A549 cells were treated with compound **3d** at 2.5 μM and 5 μM for 12 h, 24 h or 48 h. Western blot analysis (**B**). The relative levels of cleaved-PARP were normalized by the level of β-actin, and represented as percent of control. The control group (Ctrl) was treated with 0.1% DMSO (V/V). Results are mean ± SEM (*p < 0.05, **p < 0.01 vs control. N = 3).

To detect whether compounds 3c, **3d**, 6c (2.5 μM) induced apoptosis of A549 lung cancer cells, we performed Hoechst 33258 staining assay. The data suggested that compound **3d** (2.5 μM) could notably trigger apoptosis in A549 lung cancer cells through inducing chromatin condensation and nuclear fragmentation formation (Fig. 3A). We further detected the protein level of cleaved-PARP for researching the effect of compound **3d** (2.5 μM, 5 μM) in A549 lung cancer cells. As a major member of Poly (ADP-ribose) polymerases (Parps) family, PARP plays a crucial role in modulating DNA repair and death of cells^37^. Moreover, up-regulation of cleaved-PARP is one of the main characteristics of cell apoptosis^38^. The results showed that the level of cleaved-PARP (89 KDa) increased after treatment with compound **3d** (2.5 μM, 5 μM) (Fig. 3B). It indicated that compound **3d** induced A549 cell apoptosis obviously. In the other hand, we investigated if these compounds could induce autophagy or necrosis in A549 lung cancer cells by performing acridine orange (AO) staining assay, western blot analysis (microtubule-associated protein1 light chain 3 II, LC3-II) and LDH assay. The results demonstrated that compound **3d** did not cause autophagy or necrosis in A549 lung cancer cells (data not shown).

### Compound 3d increases oxidative stress to induce A549 lung cancer cell apoptosis

**Figure 4:**
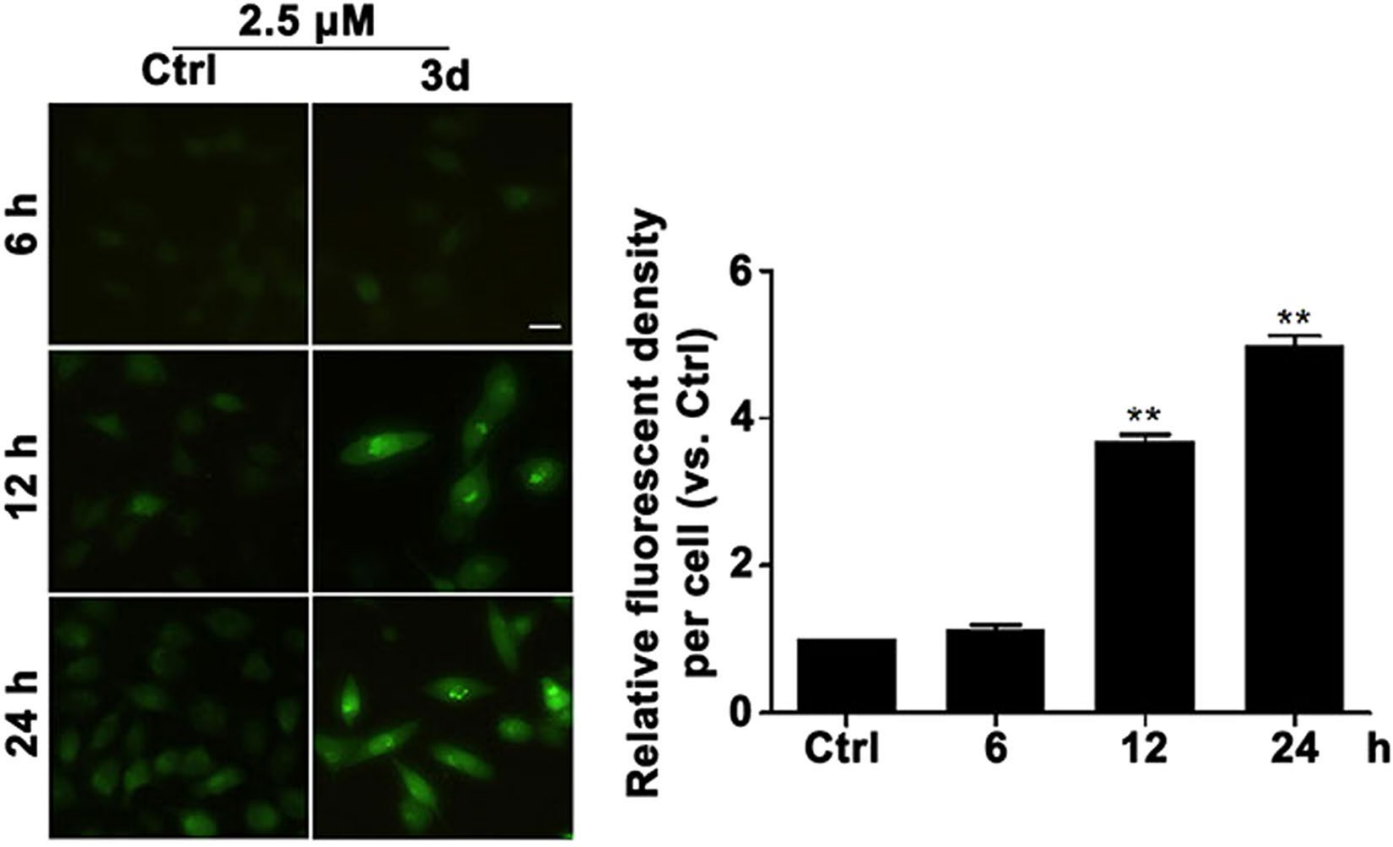
Compound **3d** increased the level of ROS in A549 lung cancer cells. The level of ROS was detected after the cells were treated with compound **3d** at 2.5 μM for 6 h, 12 h and 24 h (magnification 200×). Fluorescent image of A549 cells stained by DCHF-DA. Fluorescence quantitative statistics. Scale bar: 10 μm. Data are mean ± SEM (**p < 0.01 vs. control. N = 3).

It has been reported that oxidative stress can alter the redox balance in tumor microenvironments and impact metabolic pathways in cancer cells^39^. Cancer cells are vulnerable to high levels of reactive oxygen species (ROS)^40^. Evidence indicates that ROS influences proliferation and apoptosis in various cancers^41^. The overproduction of ROS results in oxidative stress and induces in cell apoptosis^42^. Therefore, we detected the level of ROS after treatment with compound **3d** (2.5 μM) for 12 h and 24 h. The data demonstrated that **3d** time-dependently increased the level of ROS (Fig. 4).

### Compound 3d induces Nrf-2 nuclear translocation in A549 lung cancer cells

**Figure 5:**
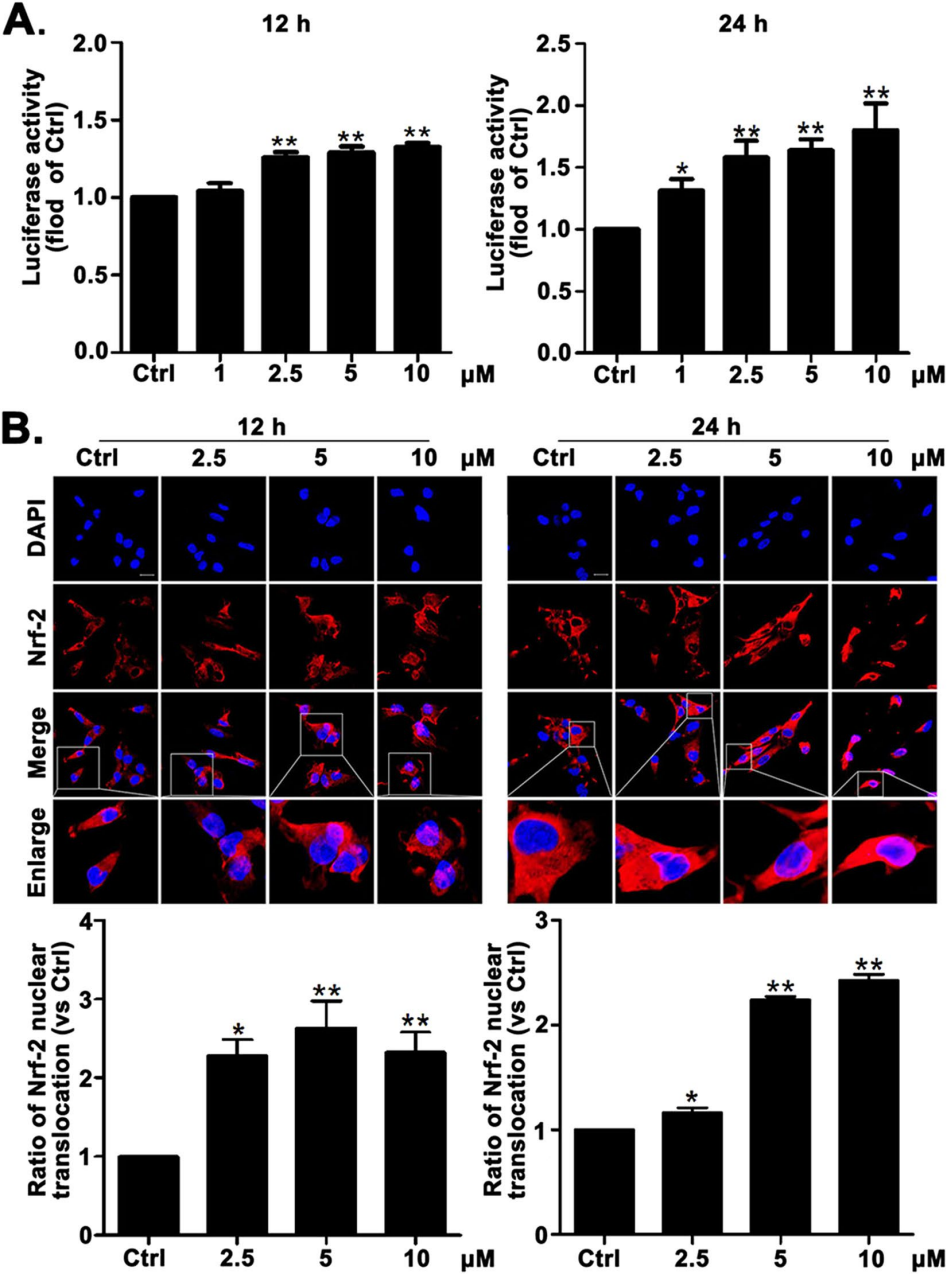
Effects of compound 3d on Nrf-2 activity and nuclear translocation. HeLa cells which contain Nrf-2-responsive/pGL4-3× ARE-basic luciferase reporter vector were treated with compound **3d** at 1, 2.5, 5, 10 μM for 12 h or 24 h. The control group (Ctrl) was treated with 0.1% DMSO (V/V). Luciferase activity was determined by luciferase assay, and normalized to cell viability measured by SRB assay (**A**). Immunofluorescence staining showing Nrf-2 nuclear translocation increased after treated with compound **3d** at 2.5, 5, 10 μM for 12 h or 24 h (**B**). Scale bar: 20 μm. Results are mean ± SEM (*p < 0.05, **p < 0.01 vs control. N = 3).

Based on our previous research results, we combined Nrf-2 activity with immunofluorescence analysis to investigate whether compound **3d** could induce Nrf-2 nuclear translocation. The luciferase assay suggested that compound **3d** elevated Nrf-2 activity significantly in dose dependent manner after treatment for 12 h or 24 h (Fig. 5A). In addition, immunofluorescence analysis suggested that compound **3d** promoted Nrf-2 nuclear translocation conspicuously (Fig. 5B).

### Compound 3d enhances the expression of HO-1 in A549 lung cancer cells

**Figure 6:**
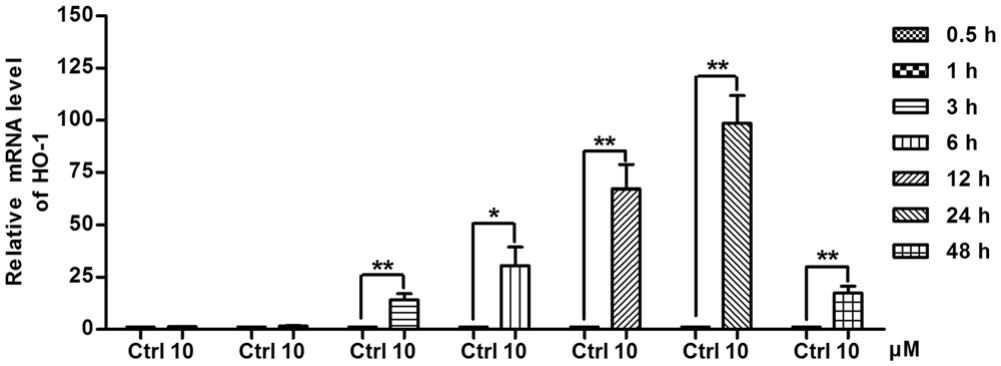
Effects of compound **3d** on the mRNA level of HO-1. A549 lung cancer cells were treated with compound **3d** at 10 μM for 0.5, 1, 3, 6, 12, 24, 48 h. The relative mRNA level of HO-1 was detected by quantitative reverse transcription–polymerase chain reaction (qRT-PCR). Results are mean ± SEM (*p < 0.05, **p < 0.01 vs control. N = 3).

It has been reported that the oxidative stress could induce HO-1 gene transcription in tumor cells through activation of Nrf-2^43^. Therefore, we detected the mRNA level of HO-1 after treatment with compound **3d** (10 μM) for 0.5, 1, 3, 6, 12, 24, 48 h. The data revealed that compound **3d** elevated the expression of HO-1 remarkably in A549 lung cancer cells (Fig. 6).

### Compound 3d inhibits the tumor xenograft growth in the chickembryo chorioallantoic membrane (CAM) model

**Figure 7:**
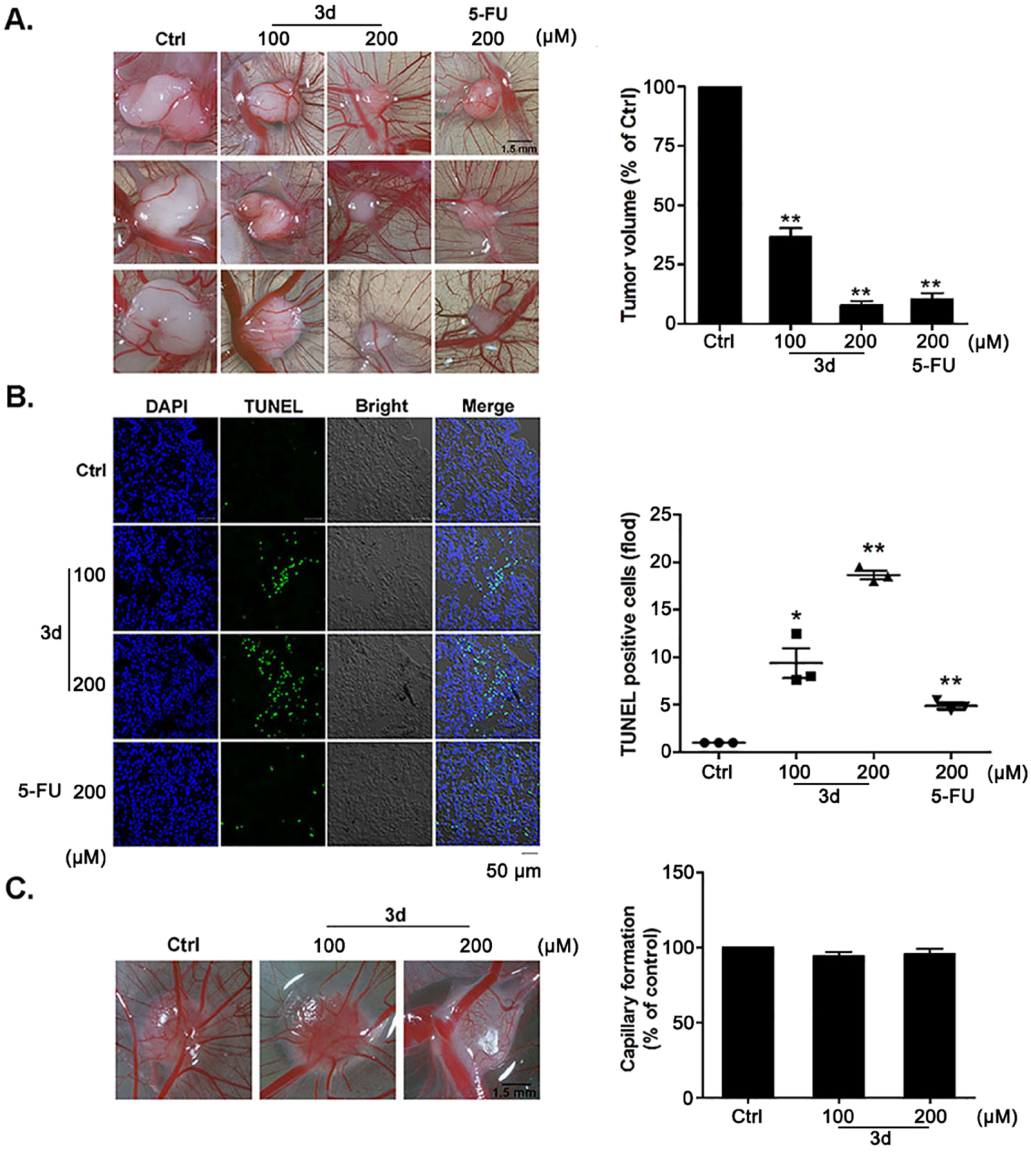
Effects of 3d on the tumor xenograft growth and normal CAM angiogenesis *in vivo*. Images of control and treated tumors photographed by biomicroscopy (**A**). Tumor volume was quantified. The volume of **3d**-treated tumor is smaller than control group. Bar, 1.5 mm, N = 5. TUNEL staining analysis of the frozen sections of tumors treated differently, and apoptotic rate was quantified (**B**). **3d** (100 μM, 200 μM) induced tumors’ aponptosis most obviously compared to the treatment of control (0.1% DMSO) and 5-FU (200 μM). Bar, 5 μm, N = 5. Angiogenesis on gelatin sponge with the treatment of **3d** or DMSO (control) photographed by biomicroscopy and quantified (**C**). The results showed that **3d** did not affect capillary formation. Bar, 1.5 mm, N = 5. Data are mean ± SEM (*p < 0.05, **p < 0.01 vs control. N = 5).

We further investigated whether compound **3d** could inhibit tumor growth effectively *in vivo*. The chick embryo chorioallantoic membrane (CAM) has been widely used in studying tissue grafts, tumor growth, angiogenic or toxicological analysis as the chick’s immunocompetent system and the immune rejection are not fully developed^44^. Therefore, we investigated the effect of **3d** on tumor growth and normal angiogenesis by CAM model. 5-FU was used for positive control drug. After seeding A549 lung cancer cells on the CAM surface for 2 days, the tumor tissue masses formed locally. Then from the day 3 to 8, we treated the tumor tissue masses with PBS, PBS/**3d** or PBS/5-FU every 2 days. The results demonstrated that **3d** significantly suppressed tumor growth (Fig. 7A). TUNEL assay was used to detect whether **3d** could induce cell apoptosis in solid tumor. After the TUNEL staining and confocal microscopy analysis of frozen sections of tumors, we discovered that **3d** could promote tumor apoptosis significantly *in vivo* (Fig. 7B). We further determined the effect of **3d** on normal CAM angiogenesis. The data revealed that **3d** had no negative effect on CAM normal angiogenesis (Fig. 7C). Therefore, *in vivo*, **3d** could inhibit tumor growth effectively by inducing apoptosis without affecting CAM normal angiogenesis.

### Compound 3d does not induce cell cycle arrest of A549 lung cancer cells

We also explored if compound **3d** had any effects on cell cycle of A549 lung cancer cells. The flow cytometry analysis showed that after being treated with **3d** (2.5 μM) for 48 h, the cell cycle of A549 lung cancer cells has not been arrested (Supplementary Figure 1).

## Discussion

Apoptosis or programmed cell death is a natural way of removing cells which are under the circumstance of pathology or aging from the body. There are a lot of anti-cancer therapies triggering apoptosis induction to kill malignant cells. However, long-term treatment with certain drugs might induce a decline of drug sensitivity in cancers which is caused by resistance^45^. In order to settle with therapy resistance in cancers, exploring new drugs to resist tumors is urgent. More importantly, for the purpose of promoting the development of new therapies for cancer or other human diseases, targeting apoptosis regulators is an attractive strategy^46^. Our data indicated that compound 3d inhibited the growth of A549 cells effectively through the way of causing apoptosis *in vitro* (Fig. 3) and *in vivo* (Fig. 7B).

In addition, compound **3d** increased the level of ROS (Fig. 4) which is accompanied by Nrf-2 activation (Fig. 5A). As a redox sensitive transcription factor, Nrf-2 plays an important role in antioxidant defense and protects cells from oxidative stress injury^47^. It has been reported that HO-1 is one of the stress response genes which is regulated by Nrf-2 through consensus cis-elements called ARE^48^. Therefore, we next detected the mRNA level of HO-1 and the data suggested that **3d** increased the expression of HO-1 (Fig. 6) in a time-depended manner.

We hypothesized that the apoptosis of A549 lung cancer cells induced by compound **3d** may be due to its strong oxidative stress injury to cells, which lead to the activation and nucleus transportation of Nrf-2. In addition, the expression of antioxidant gene HO-1 increased prominently. However, the system of its own antioxidant stress system was difficult to resist compound **3d** induced oxidative stress injury, and ultimately caused programmed cell death of A549 cells. This is the possible mechanism by which compound **3d** induced A549 cells apoptosis. It has been reported that Nrf-2 is bound to Keap1 under normal conditions. While the balance of intracellular reactive oxygen species is broken and oxidative stress occurs, Nrf-2 is separated from Keap1 and transports to nucleus^32^. Therefore, in other situations, **3d** may influence the interaction between Nrf-2 and Keap1 by suppressing Keap1. However, the exact mechanism of compound **3d** inducing A549 cells apoptosis is necessary to be investigated in our next study.

## Conclusion

After treatment with a series of novel substituted phenyl-(3-methyl-1H-indol-2-yl) -prop-2-en-1-one compounds, we observed a dose-dependent and time-dependent inhibition of growth in A549 lung cancer cells. Among the nine indolyl-chalcone derivatives, compound **3d** inhibited the viability of A549 lung cancer cells obviously through inducing apoptosis and activating Nrf-2/HO-1 pathway. *In vivo*, **3d** performed anti-growth activity of the tumor in an avian embryo model effectively and there was no negative effect on normal CAM angiogenesis. It indicates that the novel indolyl-chalcone derivative **3d** possesses great potential as an activator of Nrf-2 in cancer therapy.

## Materials and Methods

### Ethics statement

All experimental procedures and animal care in this work were performed in accordance with the ARRIVE guidelines 39 and approved by the ethics committee in Shandong University.

### Apparatus and chemicals

^1^H NMR (300 MHz or 400 MHz) and ^13^C NMR (75 MHz or 100 MHz) spectra were acquired on a Bruker Avance 300 spectrometer or Bruker Avance 400 spectrometer, with *d*6-DMSO or *d*6-Acetone used as a solvent and tetramethylsilane (TMS) as an internal standard. High resolution mass spectrometry (HRMS) involved a Q-TOF6510 spectrograph (Agilent). Unless otherwise stated, all reagents were used without further purification from merchants. Twice-distilled water was used throughout all experiments.

### Cell culture

Human lung cancer cell line A549 grew in RPMI-1640 medium (Gibco, 3180-022) containing with 10% (V/V) bovine calf serum. HeLa cells which were transfected with luciferase-based Nrf-2 reporter plasmid were grown in Dulbecco’s modified Eagle’s medium (DMEM, Gibco, 12800-058) with 10% bovine calf serum. All cell lines were cultured at 37 °C in humidified air with 5% CO_2_. Cells were seeded in 24 well plates or other appropriate dishes (30000 cells/ml).

### Cell viability assay (SRB)

A549 lung cancer cells were cultured onto 96 well plates as previously described. Next, treating cells with 0.1% DMSO or compounds 3a-**3d**, 6a-6e and 5-FU at 0.1, 1, 2.5, 5, 10 μM for 24 h or 48 h. Cell viability was analyzed by Sulforhodamine B (SRB, Sigma-Aldrich, USA) assay according to the manufacturer’s instructions.

### Western blotting

Cells were washed twice with PBS and lysed in 100 μl protein lysis buffer (Shanghai beyotime Co., China). All cell lysates were centrifuged at 12,000 × g for 15 min by using a refrigerated centrifuge. Then the protein concentrations were analyzed by using bicinchoninic acid (BCA) protein assay kit (Beyotime Co, China). After SDS-PAGE at 4 °C for 2 h, transferred to PVDF membranes (Millipore, USA). At room temperature, the membrane was blocked with 5% non-fat milk in TBST (TBS containing 0.05% Tween 20) for 1 h. Thereafter the membrane was incubated with anti-PARP (Cell Signaling, Beverly, MA, USA) and anti-β-actin (Santa Cruz Biotechnology, Dallas, TX, USA) antibodies overnight at 4 °C, washed 3 times with TBST for 5 min. Subsequently incubated with secondary antibodies which are HRP-conjugated for 1 h at room temperature. The membrane was incubated with HRP substrate for 4 min after washed 3 times with TBST and the fluorescence signals were detected by using X-ray films. The protein was quantified using Image J software.

### Hoechst 33258 staining

A549 lung cancer cells grew on 24 well were stained with 10 mg/ml Hoechst 33258 and avoid the light for 30 min at 37 °C after treatment with 0.1% DMSO or compounds 3c, **3d**, 6c for 24 h and 48 h. Cells were washed with PBS for twice then photographed by using an Olympus (Japan) BH-2fluorescence microscope.

### Measurement of intracellular ROS

As previously described, A549 lung cancer cells grew on 24 well were washed with RPMI-1640 medium for 5 min and incubated with 10 μM 2′, 7′-dichlorodihydrofluorescein (DCHF, Sigma-Aldrich) at 37 °C for 30 min. After washed 3 times with PBS, it was photographed by utilizing an Olympus (Japan) BH-2fluorescence microscope.

### Luciferase assay

HeLa cells which contain Nrf-2-responsive**/**pGL4-3 × ARE-basic luciferase reporter vector were seeded onto 96-well plates and cultured overnight, then incubated with indolyl-chalcones derivatives (**3d**) at the different concentrations (1, 2.5, 5, 10 μM) and times (12 h or 24 h). Luciferase activity of cells was examined by using Luciferase Reporter Gene Assay Kit (Beyotime, China) and normalized to cell viability measured by Sulforhodamine B (SRB) assay.

### Immunofluorescence Assay

Immunofluorescence assay was performed as described^49^. In brief, A549 lung cancer cells were fixed with 4% paraformaldehyde for 15 min and blocked with 3% normal donkey serum (Solarbio, SL050) for 20 min at room temperature. Then, the cells were incubated with primary antibody (1:100) (Nrf-2, Proteintech, America) at 4 °C overnight and then corresponding secondary antibody (1:200) at 37 °C for 1 h. Cells were washed 3 times with 0.1 M phosphate-buffered saline (PBS; 137 mM NaCl, 2.7 mM KCl, 10 mM Na_2_HPO_4_, and 2 mM KH_2_PO_4_). Cells were incubated with DAPI for 10 min and washed 3 times with PBS, then photographed by using confocal fluorescence microscopy Zeiss LSM700 (Germany).

### Quantitative real-time PCR

Extraction of total RNA use of Trizol reagent (Invitrogen, USA). The reverse transcription step involved use of the PrimeScript RT reagent kit with gDNA Eraser (DRR047, TAKARA). The relative mRNA level of HO-1 was quantified by SYBR Premix Ex Taq (Tli RNaseH Plus) RT-PCR reactions. The expression of β-actin was used to normalize with a melting curve for each reaction. Primers for HO-1 were: sense TGCACATCCGTGCAGAGAAT; antisense CTGGGT TCTGCTTGCTTGTTTCGC. Primers for β-actin were: sense GAAGTGTGACGTGGACATCC; antisense CCGATCCACACGGAGTACTT.

### *In vivo* tumor assay of chick embryo chorioallantoic membrane (CAM)

The fertilized chicken eggs were incubated at 37 °C with 60% relative humidity. On embryonic day 8, a silicone ring with a 5.5 mm inner diameter was placed on the CAM, and then 8 million A549 lung cancer cells in 20 μl of medium were seeded into this silicone ring. Eggs were divided into four groups in which 5 eggs were contained. On day 3, every egg was treated with **3d** at the concentrations of 100 or 200 μM every 2 days, 5-FU (200 μM) was as the positive control group. After treatment with **3d** or 5-FU for 6 days, samples of the CAM and tumors were taken. The size of tumors were measured and the tumor volume calculation was performed as described in literature^50^.

### TUNEL assay

TUNEL assay was performed according to the manufacturer’s instructions (Promega, USA) to detect DNA fragmentation of the tumor tissues. Then the apoptosis was assessed by utilizing laser scanning confocal microscopy Zeiss LSM700 (Germany).

### Angiogenesis assay of CAM

The fertilized chicken eggs were incubated at 37 °C with 60% relative humidity. On embryonic day 9, the gelatin sponge absorbed compound **3d** (100 and 200 μM) or DMSO was placed on the CAM. The treatment with **3d** or DMSO was performed every 2 days. After 6 days, the CAM zones including the gelatin sponge were taken out. The biomicroscopy image and quantitative analysis were performed by Image-Pro Plus.

### Flow cytometric analysis

A549 cancer cells were treated with compound 3d (2.5 μM) or DMSO for 48 h, then gathered by centrifugation at 400 g, 4 °C for 5 min. Cells were fixed with 75% ethanol, then stained with 2 mg/ml propidium iodide (PI) containing 1 mg/ml RNase A at 4 °C for 30 min. The stained cells were analyzed by flow cytometry (Amnis ImageStream Mark II, USA).

### Statistical analysis

All data were presented as means ± SEM from at least three independent experiments and analyzed by SPSS (Statistical Package for the Social Sciences) software. When p value was < 0.05, differences were recognized as statistically remarkable.

## Electronic supplementary material

Supplementary Information
