# Supplementary material for: Novel indolyl-chalcone derivatives inhibit A549 lung cancer cell growth through activating Nrf-2/HO-1 and inducing apoptosis in vitro and in vivo
Source: Sci Rep. 2017 Jun 20;7:3919. doi: 10.1038/s41598-017-04411-3 (PMC5478673; doi:10.1038/s41598-017-04411-3)
Supplement: Supplementary file 1 — Supplementary Information [file 41598_2017_4411_MOESM1_ESM.pdf]

Supplementary information for:

Novel indolyl-chalcone derivatives inhibit A549 lung cancer cell growth through activating Nrf-2/HO-1 and inducing apoptosis *in vitro* and *in vivo*

Xuan Zhao[1#], WenLiang Dong[2#], YuanDi Gao[1], Dong-Shoo Shin[2], Qing Ye[3], Le Su[1], Fan Jiang[3], BaoXiang Zhao[4, *], JunYing Miao[1, 3, *]

[1]*Shandong Provincial Key Laboratory of Animal Cells and Developmental Biology, School of Life Science, Shandong University, Jinan, 250100, China*

[2]*Department of Chemistry, Changwon National University, Changwon, 51140, South Korea*

[3]*The Key Laboratory of Cardiovascular Remodeling and Function Research, Chinese Ministry of Education and Chinese Ministry of Health, Qilu Hospital, Shandong University, Jinan, 250012, China*

[4]*Institute of Organic Chemistry, School of Chemistry and Chemical Engineering, Shandong University, Jinan, 250100, China*

* Correspondence to:

Prof. BaoXiang Zhao, Institute of Organic Chemistry, School of Chemistry and Chemical Engineering, Shandong University, Jinan, 250100, China.

Fax: + 86 531-88564464; Tel.: + 86 531 88366425.

E-mail address: bxzhao@sdu.edu.cn

Prof. JunYing Miao, Shandong Provincial Key Laboratory of Animal Cells and Developmental Biology, School of Life Science, Shandong University, Jinan, 250100, China.

Fax: + 86 531 88565610; Tel.: + 86 531 88364929.

E-mail address: miaojy@sdu.edu.cn

Supplementary materials and methods:

Spectroscopic data of compounds:

3a: (*E*)-3-(2-methoxyphenyl)-1-(3-methyl-1H-indol-2-yl) prop-2-en-1-one

^{1}H NMR (Acetone-d6, 300 MHz): δ = 2.78 (s, 3H, CH_3), 3.99 (s, 3H, OCH_3), 7.11-7.16 (m, 3H, ArH), 7.29-7.35 (m, 1H, ArH), 7.43-7.54 (m, 2H, ArH), 7.71-7.82 (m, 2H, ArH), 7.89 (d, 1H, J = 15.6 Hz, =CH), 8.12 (d, J = 15.6 Hz, 1H, CH), 10.68 (s, 1H, NH). ^{13}C NMR (Acetone-d6, 75 MHz) δ: 11.18, 56.09, 112.44, 113.11, 118.75, 120.58, 121.68, 124.66, 125.23, 126.55, 128.84, 129.87, 130.03, 132.66, 134.46, 137.80, 138.50, 159.79, 183.07; HRMS calcd for $[M+H]^+$ $C_{19}H_{18}NO_2$: 292.1338, found 292.1386.

3b: (*E*)-3-(4-chlorophenyl)-1-(3-methyl-1H-indol-2-yl) prop-2-en-1-one

^{1}H NMR (Acetone-d6, 300 MHz): δ = 2.77 (s, 3H, CH_3), 7.10-7.16 (m, 1H), 7.30-7.36 (m, 1H, ArH), 7.50-7.55 (m, 3H, ArH), 7.74-7.87 (m, 5H, ArH), 10.74 (s, 1H, NH). ^{13}C NMR (Acetone-d6, 75 MHz) δ: 11.12, 113.14, 119.57, 120.69, 121.74, 125.50, 126.77, 129.77, 129.99 (2H), 130.85 (2H), 134.06, 135.03, 136.38, 137.91, 141.62, 182.57; HRMS calcd for $[M+H]^+$ $C_{18}H_{15}ClNO$: 296.0842, found 296.0863.

3c: 3-(2, 3-dichlorophenyl)-1-(3-methyl-1H-indol-2-yl) prop-2-en-1-one

^{1}H NMR (DMSO-d6, 400 MHz): δ = 2.67 (s, 3H, CH_3), 7.11 (t, J = 7.6 Hz, 1H), 7.34 (t, J = 7.6 Hz, 1H), 7.46-7.54 (m, 2H, ArH), 7.72-7.76 (m, 2H, ArH), 7.88 (d, 1H, J = 15.6 Hz, =CH), 8.00 (d, 1H, J = 15.6 Hz, =CH), 8.05-8.07 (m, 1H, ArH), 11.71 (s, 1H, NH). ^{13}C NMR (DMSO-d6, 100 MHz) δ: 11.12, 112.99, 120.16, 120.28, 121.41, 126.55, 127.38, 128.53, 128.60, 129.02, 132.30, 132.38, 132.89, 133.15, 135.66, 137.19, 137.32; HRMS calcd for $[M+H]^+$ $C_{18}H_{14}Cl_2NO$: 330.0452, found 330.0394.

3d: 1, 1'-(3-methyl-1H-indole-1, 2-diyl) bis (3-phenylprop-2-en-1-one)

^{1}H NMR (Acetone-d6, 300 MHz): δ = 2.61 (s, 3H, CH_3), 3.99 (s, 3H, CH_3), 7.09-7.14 (m, 1H), 7.29-7.35 (m, 1H), 7.47 (d, J = 8.4 Hz, 1H, ArH), 7.62-7.69 (m, 2H), 8.13 (d, J = 15.6 Hz, 1H, =CH), 8.35-8.43 (m, 4HH); ^{13}C NMR (Acetone-d6, 75 MHz) δ: 10.86, 31.19, 110.74, 118.93, 120.57, 120.71, 121.37, 124.64(2C), 125.68, 128.93, 130.41(2C), 132.64, 134.16, 140.24, 144.26, 188.34; HRMS calcd for $[M+H]^+$ $C_{27}H_{22}NO_2$: 392.1651, found 392.1615.

6a: 1-(4-bromophenyl)-3-(1, 3-dimethyl-1H-indol-2-yl) prop-2-en-1-one

^{1}H NMR (Acetone-d6, 300 MHz): δ = 2.45 (s, 3H, CH_3), 3.83 (s, 3H, CH_3), 6.94-7.00 (m, 1H), 7.14-7.19 (m, 1H, ArH), 7.32 (d, J = 8.4 Hz, 1H, ArH), 7.44-7.54 (m, 2H), 7.61-7.66 (m, 2H), 7.92-7.98 (m, 3H); ^{13}C NMR (Acetone-d6, 75 MHz) δ:10.80, 31.15, 110.66, 118.05, 120.46, 120.57, 121.61, 125.36, 127.87, 128.96, 131.09 (2C),

132.77 (2C), 133.27, 138.38, 140.04, 188.39; HRMS calcd for $[M+H]^+$ $C_{19}H_{17}BrNO$: 354.0494, found 354.0457.

6b: 3-(1, 3-dimethyl-1H-indol-2-yl)-1-(4-nitrophenyl) prop-2-en-1-one

^{1}H NMR (Acetone-d6, 400 MHz): δ = 2.60 (s, 3H, CH_3), 3.97 (s, 3H, CH_3), 7.10 (t, J = 7.6 Hz, 1H, ArH), 7.31 (t, J = 7.6 Hz, 1H, ArH), 7.46 (d, J = 8.4 Hz, 1H, ArH), 7.60 -7.67 (m, 2H), 8.12 (d, J = 15.6 Hz, 1H, =CH), 8.34-8.41 (m, 4H); ^{13}C NMR (Acetone-d6, 100 MHz) δ: 9.96, 30.31, 109.85, 118.05, 119.69, 119.83, 120.55, 123.74 (2C), 124.80, 128.07, 129.52 (2C), 131.78, 133.29, 139.38, 143.40, 150.17, 187.49; HRMS calcd for $[M+H]^+$ $C_{19}H_{17}N_2O_3$: 321.1239, found 321.1185.

6c: 1-(2, 5-dihydroxyphenyl)-3-(1, 3-dimethyl-1H-indol-2-yl) prop-2-en-1-one

^{1}H NMR (Acetone-d6, 300 MHz): δ = 2.63 (s, 3H, CH_3), 4.00 (s, 3H, CH_3), 6.88 (d, J = 9.0 Hz, 1H), 7.09-7.15 (m, 2H, ArH), 7.29-7.34 (m, 1H, ArH), 7.47 -7.54 (m, 2H, ArH), 7.62-7.69 (m, 2H), 8.16 (s, 1H), 8.21 (s, 1H), 12.45 (s, 1H); ^{13}C NMR (Acetone-d6, 75 MHz) δ:10.09, 109.86, 114.08, 117.89, 118.67, 118.77, 119.47, 119.54, 119.66, 119.81, 124.68, 124.77, 128.07, 131.82, 132.84, 139.30, 149.35, 156.98, 192.80; HRMS calcd for $[M+H]^+$ $C_{19}H_{18}NO_3$: 308.1287, found 308.1291.

6d: 3-(1-benzyl-3-methyl-1H-indol-2-yl)-1-(4-bromophenyl) prop-2-en-1-one

^{1}H NMR (Acetone-d6, 300 MHz): δ = 2.64 (s, 3H, CH_3), 5.71 (s, 2H, CH_2), 7.13-7.19 (m, 3H), 7.26-7.43 (m, 5H, ArH), 7.50 (d, J = 8.4 Hz, 1H, ArH), 7.68-7.75 (m,

3H), 7.82 -7.86 (m, 2H), 8.20(d, J = 15.6 Hz, 1H, =CH); ^{13}C NMR (Acetone-d6, 75 MHz) δ:10.82, 47.79, 110.88, 118.94, 120.78, 120.92, 121.82, 125.78, 126.92 (2C), 127.83, 128.23, 129.18, 129.71 (2C), 130.91 (2C), 132.48, 132.69 (2C), 132.93, 138.19, 139.32, 140.16, 188.22; HRMS calcd for $[M+H]^+$ $C_{25}H_{21}BrNO$: 430.0807, found 430.0755.

6e: 3-(1- ((6-chloropyridin-3-yl) methyl)-3-methyl-1H-indol-2-yl)-1-(4-nitrophenyl) prop-2-en-1-one

^{1}H NMR (DMSO-d6, 400 MHz): δ = 2.61 (s, 3H, CH_3), 5.77 (s, 2H, CH_2), 7.15 (t, J = 7.2 Hz, 1H), 7.29-7.36 (m, 2H), 7.43-7.51 (m, 2H, ArH), 7.63(d, J = 8.4 Hz, 1H, ArH), 7.74 (d, J = 8.4 Hz, 1H, ArH), 7.96 (d, J = 15.6 Hz, 1H, =CH), 8.19-8.24 (m, 3H), 8.36 (d, J = 8.4 Hz, 2H, ArH); ^{13}C NMR (DMSO-d6, 100 MHz) δ:11.34, 43.84, 110.86, 119.24, 120.66, 120.80, 121.96, 124.36, 124.94 (2C), 125.75, 128.44, 130.12, 131.26 (2C), 133.33, 134.03, 138.11, 139.00, 143.10, 148.32, 149.74, 150.24, 188.12; HRMS calcd for $[M+H]^+$ $C_{24}H_{19}ClN_3O_3$: 432.1115, found 432.1125.

Supplementary Figures and Figure legends:

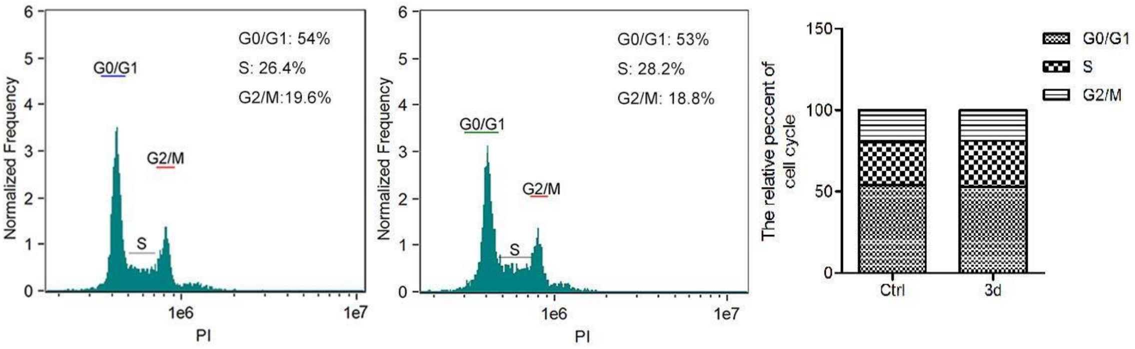

Supplementary Figure 1. **Effect of compound 3d on the cell cycle of A549 lung cancer cells.** After being treated with **3d** (2.5 μM) for 48 h, apoptosis was determined by flow cytometry analysis (Amnis ImageStream MarkⅡ, USA).

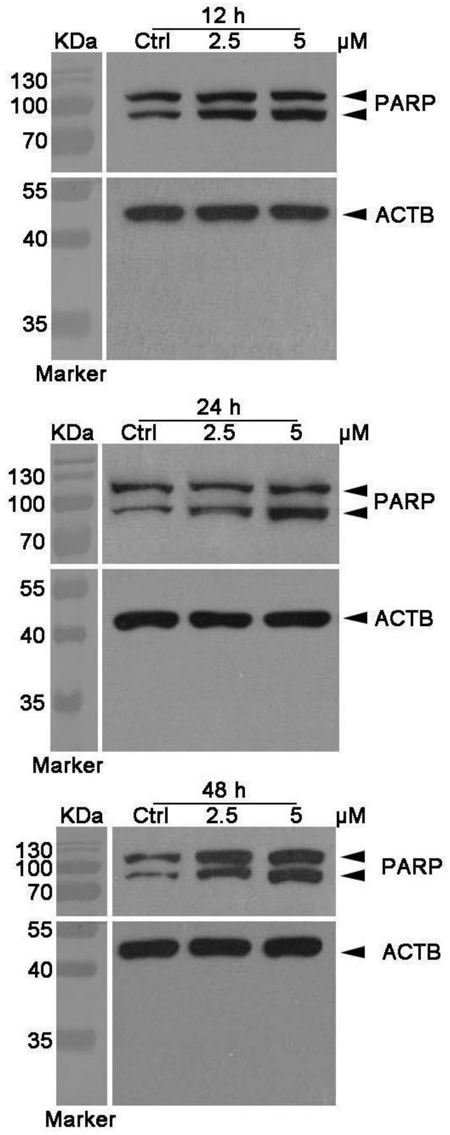

Supplementary Figure 2. **Uncropped blots probed with PARP and ACTB.**
